# Evolving challenges in the management of early esophageal adenocarcinoma: from clinical staging to molecular insights

**DOI:** 10.1186/s12957-026-04285-8

**Published:** 2026-03-10

**Authors:** Lorenzo Giorgi, Giovanni Maria Garbarino, Renato De Martino, Andrea Pansa, Marta Casiraghi, Silvia Basato, Rita Alfieri, Carlo Castoro

**Affiliations:** 1 Upper Gastrointestinal Surgery Unit, IRCCS Humanitas Research Hospital, Rozzano, Milan, 20089 Italy; 2 Department of Biomedical Sciences, Humanitas University, Pieve Emanuele, Milan, 20072 Italy

## Abstract

This report explores the evolving management of early esophageal adenocarcinoma (EAC), focusing on the therapeutic balance between endoscopic resection and esophagectomy. It synthesizes recent evidence from international guidelines, surgical and endoscopic series, and predictive modeling studies to clarify lymph-node metastasis (LNM) risk, evaluate current staging limitations, and discuss future directions in treatment stratification and personalized care.

## Introduction

Over the past two decades, the widespread adoption of surveillance endoscopy for Barrett’s esophagus (BE) has led to an increase in the detection of early esophageal adenocarcinoma (EAC). [1, 2] Simultaneously, the refinement of endoscopic mucosal resection (EMR) and endoscopic submucosal dissection (ESD) techniques has shifted the therapeutic paradigm, allowing organ-preserving treatment for superficial lesions that once mandated esophagectomy. [3, 4] However, enthusiasm for endoscopic innovation must be measured against rigorous risk assessment: in early-stage EAC, treatment decisions hinge on the probability of lymph-node metastasis (LNM), the principal determinant of long-term prognosis. [5] The clinical relevance of early EAC is amplified by epidemiologic trends. According to U.S. Surveillance, Epidemiology, and End Results (SEER) data, the incidence of EAC has increased sixfold since the 1970s, now exceeding 6 cases per 100,000 person-years in the US and Northern Europe. [6, 7] This rise mirrors the growing prevalence of BE, which affects 1–2% of the general population and carries a 0.54% annual risk of progression to EAC progression. [8] As detection at potentially curable stages becomes more common, the challenge lies in selecting the optimal therapeutic path. [9] This review synthesizes current guideline definitions, risk stratification frameworks, and treatment strategies for early EAC. A comprehensive literature search was performed across PubMed, Scopus, and Google Scholar for English-language studies published between 2000 and 2025. The primary search query on PubMed yielded an initial pool of 857 records, which served as the foundational dataset for screening. Scopus and Google Scholar were further utilized for cross-referencing and to identify additional evidence integrating the search findings through backward and forward snowballing. From this combined search, titles and abstracts were screened to prioritize the highest levels of evidence, including international guidelines, meta-analyses, and foundational narrative reviews. Furthermore, the core of our discussion is supported by original clinical research, with an emphasis on high-volume multicenter cohorts, propensity-matched analyses, and large-scale population registries.

### Definition and guideline variability: same disease, different treatment algorithm

The term *early EAC* refers to tumors confined to the mucosa (T1a) or submucosa (T1b) without regional or distant metastases (N0M0). However, its clinical interpretation and, crucially, the threshold for endoscopic versus surgical treatment, varies across international guidelines due to differing definitions of “low risk” submucosal invasion.

According to the ESMO and ESGE guidelines, endoscopic resection (ER) is considered curative for all T1a and for T1b lesions with superficial submucosal invasion (SM1 ≤ 500 μm), provided that there is no lymphovascular invasion (LVI), poor differentiation (G3), or ulceration. [10, 11] This 500 μm threshold is grounded in surgical series reporting a 0–2% incidence of lymph-node metastasis (LNM) for these tumors, lower than the perioperative mortality of esophagectomy in high-volume centers [12, 13].

T1a tumors are generally considered low risk, although adverse histopathologic features can markedly increase the likelihood of nodal metastasis. [14] Small retrospective cohorts have reported nodal involvement rates approaching 20% in some series. [15–17] By contrast, high-risk T1b lesions, defined by deeper submucosal invasion and adverse features, are associated with nodal metastasis rates of 30% or higher, depending on the combination and severity of risk factors. For these patients, the ESMO guidelines recommend surgical resection with lymphadenectomy as the standard of care.

The NCCN Guidelines adopt a more qualitative approach. ER may be appropriate for mucosal or superficially submucosal invasive lesions if en bloc R0 resection is achieved and no adverse histology is present (LVI, poor differentiation, or positive deep margin). [18] However, esophagectomy remains the preferred option for all T1b tumors in fit patients, with no quantitative depth thresholds specified. [18] In contrast, the Japanese Esophageal Society provides a more granular classification: SM1 is defined as < 200 μm invasion, SM2 as 200–500 μm, and SM3 as > 500 μm. Only SM1 lesions without additional risk factors qualify for curative ESD. [19] This stricter threshold reflects Japan’s experience with squamous cell carcinoma, known for higher nodal risk, and the technical expertise in ER.

Despite differing morphometric criteria, all guidelines are based on the same biological premise: deeper invasion correlates with increased nodal metastasis: European and Japanese frameworks represent differing tolerances for oncologic risk rather than fundamentally divergent philosophies [20, 21].

Ultimately, these differing morphometric criteria represent a global lack of consensus on what constitutes “acceptable risk.” The fact that high-risk T1b lesions can reach high LNM rates underscores the limitations of current staging. The persistent “grey zone” of SM1/SM2 lesions, where treatment choice can drastically alter a patient’s quality of life, highlights the urgent need for more sophisticated diagnostic tools.

### Pathologic predictors of lymph-node metastasis: where biology exposes the hidden risk

Lymph-node metastasis (LNM) is the single most decisive prognostic factor in early EAC, directly influencing survival and therapeutic strategy. Although tumor invasion depth is strongly correlated with metastatic risk, it functions only as one dimension of a broader histopathologic risk profile.

Pooled analyses estimates the risk of LNM at approximately 4% for T1a tumors and 20–25% for T1b tumors, with stratification b submucosal invasion revealing rates near 16% for superficial involvement (SM1–SM2) and close to 30% for deeper invasion (SM3). [22] These data, however, are derived from heterogeneous cohorts and often lack adjustment for additional high-risk features, which can explain the variability observed across studies.

Among all histological parameters, LVI has emerged as the strongest and most consistent predictor of occult nodal metastasis, surpassing the prognostic value of depth alone. Superficial T1b lesions with LVI exhibit LNM rates comparable to more deeply invasive tumors meaning that biologic aggressiveness cannot be inferred from morphometric depth in isolation. [23] Moreover, recent works suggest that not only the presence but also the *extent* of LVI is clinically relevant, with metastatic risk rising sharply as the number of LVI foci increases [24].

These findings are supported by the underlying anatomy of the esophageal wall, which contains a dense lymphatic plexus within the lamina propria and muscularis mucosae. This anatomic arrangement facilitates lymphatic access even in intramucosal tumors. Classic anatomic studies demonstrated that lymphatic channels traverse the lamina propria, providing a potential substrate for nodal spread even in T1a disease. [25] Subsequent morphometric analyses revealed significant interindividual variability in lymphatic vessel density and architecture, up to a two- to threefold difference in both density and length, which may partly explain why some superficially invasive tumors metastasize while others do not [26].

In addition to LVI and depth, tumor differentiation and size independently influence nodal risk. [23] Poorly differentiated tumors (G3) are associated with LNM rates as high as 20%, compared to 2% in well- or moderately differentiated lesions. [27] Tumor size also shows a clear correlation: lesions ≥ 2 cm carries a significantly higher risk of nodal involvement, with LNM rates ranging from 18% to 36%, in contrast to < 5% in tumors < 2 cm. Recent population-level analyses corroborate these findings, identifying tumor size ≥ 20 mm as an independent risk factor. [27, 28] To refine individual risk prediction, several clinical nomograms and composite models have been developed. These tools incorporate combinations of depth, LVI, grade, and size, and have shown good discriminatory performance in estimating the probability of occult nodal disease. [29, 30] Taken together, these data indicate that nodal risk in early EAC emerges from a complex and nonlinear interplay of pathologic features.

### Clinical staging and the role of EUS and PET/CT: what we see and what we miss

Accurate staging is essential in early EAC, yet the diagnostic tools available fall short of resolving the subtle distinctions that determine eligibility for organ-preserving therapy. Endoscopic visualization provides an essential first assessment, but surface morphology is an unreliable surrogate for invasion depth. Even lesions that appear confined to the mucosa may harbor submucosal infiltration, leading to both over- and understaging in seemingly straightforward cases [31–33].

Endoscopic ultrasound (EUS) although widely used for locoregional staging, is constrained by the physical limits of ultrasonographic resolution in early disease. In a surgical cohort of 107 patients with cTis–cT1 disease, EUS correctly distinguished T1a from T1b lesions in only approximately half of cases; understaging occurred in up to 49% of T1b cancers, while overstaging affected nearly one-third of mucosal tumors. [34, 35]Nodal evalutation is similarly imperfect: among lesions staged as cN0, 10% harbored pathologic nodal metastases, with nodal involvement present in 17.5% of classified endoscopically as T1a. [34] Even when morphologic criteria suggest benignity, small metastatic nodes frequently escape detection, and EUS-guided FNA has limited utility in early-stage disease because targetable nodes are often subcentimetric [36, 37].

FDG–PET/CT offers whole-body metabolic assessment but performs poorly for regional nodal staging in EAC. Sensitivity for detecting metastatic regional lymph nodes range from 41 to 60% with specificities of 77–89%, with false negatives particularly common in subcentimetric or peritumoral nodes, precisely the patterns expected in early disease. [38–40] In signet-ring cell and highly mucinous adenocarcinoma FDG uptake is often minimal, reducing the sensitivity for nodal metastases to approximately 20%. These limitations render PET-CT inadequate for excluding microscopic nodal involvement [40, 41].

Nonetheless, PET/CT retains a clearly defined role in staging because of its superior ability to detect distant metastases. Large-scale data show that PET-CT reveals previously unrecognized M1 disease in 14.9% of patients deemed metastasis-free on CT, modifies clinical management in 23%, and prevents futile surgical exploration by detecting occult dissemination. [42] When incorporated into staging algorithms, PET-CT increase sensitivity for M1 disease from 37% with CT/EUS alone to 78%, correctly upstaging 20% of patients. [32]Accordingly, major guidelines endorse PET-CT prior to any curative-intent therapy, not for precise locoregional staging, but to safeguard against inappropriate surgical or multimodal intervention [11, 43].

### Surgical management and outcomes: identifying patients who benefit from esophagectomy in early EAC

Long term outcomes after ER and esophagectomy in early EAC demonstrates a stage-dependent pattern. In cT1aN0 EAC, a large propensity-matched analysis reported 5-year overall survival (OS) rates of 70% for ER versus 74% for esophagectomy, with a 4% rate of occult nodal involvement in surgical specimens. Similarly, a national dataset showed 5-year OS of 76.2% after ER and 80.2% after surgery, both substantially superior to chemoradiotherapy (CRT, 15.3%) [44, 45].

In contrast, cT1b tumors present a more complex scenario. The same matched study reported 5-year OS of 53% for ER and 61% for esophagectomy (*p* = 0.3), with nodal postoperative positivity rates of 15%, while a separate model adjusting for histological risk features, found significantly improved survival with surgery (58.8% vs. 72.0%). Perioperative mortality was higher after surgery (4–7%) compared to ER (1–2%) [44, 45].

Survival comparisons are influenced by treatment selection. ER is typically reserved for older, comorbid patients and in tumors < 20 mm, which may partly explain the observed survival differences. In this context, ER was associated with lower overall survival (65 vs. 78 months; HR 1.47), despite comparable cancer-specific survival (81.0% vs. 79.9%). [28] Staging inaccuracies further complicate clinical decision-making. Pathologic upstaging occurred in 17.3% of patients initially classified as T1b, with 5-year OS falling from 67.7% to 43.7% in upstaged cases. Notably, outcomes were worse among those treated with ER, underscoring the limitations of preoperative staging in early disease 46].

The long-term risk of recurrence after ER is also non-negligible. In a recent multicenter cohort of pT1b patients undergoing endoscopic resection with systematic follow-up, 30.9% developed metastatic progression at five years. [23, 47] These data underscore the limitations of histopathologic risk stratification and support a more cautious approach in managing T1b EAC.

Additional insights come from the retrospective multicenter CONGRESS study. [14] The overall prevalence in the cohort of LNM was 13.5%, with non-trivial rates even in T1a tumors (9.8%) and progressively higher rates in T1b sm1 (14.8%) and sm2–3 (17.9%) lesions. Notably, 15.3% of node-positive patients lacked any classical histopathologic risk factors highlighting the limitations of current ER-based risk stratification. ER provides excellent outcomes in selected cT1a and low risk cT1b tumors, but esophagectomy remains the most reliable curative strategy in unselected cT1b disease or in cases with uncertain risk profiles [48].

### The question of neoadjuvant therapy

Current guidelines converge in not recommending neoadjuvant therapy for early EAC. Management is directed toward endoscopic or surgical resection for cT1 disease, with systemic and multimodal treatment reserved for more advanced stages. [11, 18] However, in high-risk T1b tumors, the ESGE guideline limits systemic treatment to individualized consideration within multidisciplinary expertise.

Neoadjuvant chemoradiotherapy (NCRT) has not demonstrated benefit in clinically for early EAC. In contemporary surgical cohorts restricted to cT1b, preoperative chemoradiation was rarely used and consistently associated with inferior outcomes: in one large analysis, 5-year overall survival (OS) was approximately 49% with neoadjuvant chemoradiation versus 67% after upfront esophagectomy, with no advantage even among patients ultimately found to harbor nodal involvement or ≥pT2 disease at resection. [46] Broader early-stage datasets show similar findings: neoadjuvant chemoradiation yielded the poorest survival (OS 40 months) despite substantial pathological upstaging (39% for T and 30% for N), indicating that chemoradiation does not compensate for biologically aggressive disease.

The effects of systemic chemotherapy are well characterized in EAC. Pathological downstaging following neoadjuvant chemotherapy (NCT) occurs in approximately 44% of patients and is associated with a marked reduction in locoregional recurrence, a significant decrease in systemic metastases (19% vs. 29%), and a 57% reduction in the risk of recurrence or death compared with non-responders [49].

Downstaging is a powerful determinant of long-term prognosis, with patients who achieve a pathological stage equivalent to early disease generally approaching the outcomes of those who present with the same stage and undergo primary surgery. However, even among major responders, residual systemic risk is not entirely abolished, reflecting the limitations of pathological assessment in capturing micrometastatic dissemination. [50] This principle is supported by biologic evidence that micrometastatic nodal deposits are present in more than 30% of pathologically node-negative tumors and that circulating tumor cells can be detected perioperatively in up to 60% of patients. [49]This convergence of clinical and biological evidence underscores that even morphologically early tumors may possess systemic potential not captured by conventional staging, emphasizing the importance of accurately defining biological risk when determining the appropriate timing and role of systemic therapy.

Systemic therapy also influences outcomes after upfront resection when biologically advanced disease is unmasked. In large national cohorts of cT1–T2N0 patients unexpectedly upstaged to pT3–4 or pN+, adjuvant chemotherapy significantly improves survival, while postoperative radiation based regimen, either alone or combined with chemotherapy, does not confer benefit and may be detrimental. [51, 52]The benefit is presumably derived from clinically undetectable micrometastatic foci, in which the use of systemic therapy address this additional disease and lead to improved survival Conversely, among patients who remain truly pT1b–T2N0 after primary surgery, adjuvant therapy has not shown survival benefit. Across guidelines, no unique adjuvant modality emerges as clearly preferable once early-stage disease proves more advanced, leaving clinicians to individualize therapy within a context in which biological risk is evident, but the supporting evidence remains heterogeneous.

### Future directions and unresolved questions

Sentinel-node (SN) mapping has been explored as a strategy to individualize lymphadenectomy in high-risk T1b EAC, with prospective feasibility studies consistently demonstrating successful detection of SNs using technetium-based tracers, including hybrid 99mTc–ICG formulations. [49, 53–55] Identification rates approached 100%, with close concordance between preoperative imaging and intraoperative localization. Although most SNs were tumor-free, micrometastatic disease was identified in approximately 18% of patients across these series, while no metastases were detected in non-SN, suggesting a potential role for selective nodal assessment following endoscopic resection. Early functional outcomes were favorable, with preservation of esophageal and gastric integrity and minimal morbidity, although the long-term oncologic adequacy of this approach remains to be defined.

Artificial intelligence is emerging as a complementary tool to address persistent limitations in the clinical staging of early EAC. In endoscopic imaging, AI-based systems achieve diagnostic performance comparable to expert endoscopists and superior to non-experts; early investigations further suggest utility in estimating invasion depth, including real-time discrimination between T1a and T1b lesions. [56] Beyond optical diagnosis, experience from other gastrointestinal malignancies indicates that machine-learning models built on routine clinicopathologic variables can achieve high sensitivity for occult nodal metastasis and outperform traditional regression-based predictors. [57] Together, these developments suggest that AI may ultimately enhance both morphologic assessment and biologic risk stratification, reducing reliance on imperfect conventional staging.

Genomic analyses further challenge the assumption that early-stage EAC represents biologically indolent disease. Multi-omic profiling demonstrates that most driver events, TP53 mutation, CDKN2A loss, whole-genome doubling, chromosomal instability, structural variation, and amplifications in proliferative pathways, arise during Barrett’s esophagus and dysplasia, with progressive lesions exhibiting markedly greater genomic complexity than non-progressors despite minimal histologic divergence. [58, 59] These observations align with transcriptomic classifications identifying two major molecular subtypes: EA1, characterized by genomic instability, TP53 alterations, and immune-cold signaling, associated with poorer prognosis; and EA2, more differentiated and immune-active. [60] Notably, the molecular programs defining EA1 are already detectable in pre-invasive tissue, indicating that phenotypically early cancers may belong to biologically aggressive lineages from inception. This concept is reinforced by gene-expression analyses showing that a 59-gene signature derived from advanced adenocarcinoma is present in a subset of T1 lesions and accurately discriminates high-risk from low-risk early cancers, predicting adverse outcomes in a manner comparable to more advanced disease. [61] Collectively, these findings underscore that early EAC is not a biologically distinct or genomically quiescent entity; rather, the boundary between T1 and more advanced stages is largely morphological, and metastatic competence may be established well before overt invasion.

## Conclusion

Early esophageal adenocarcinoma remains a biologically heterogeneous disease in which conventional morphologic staging inadequately captures metastatic potential. Across pathology, imaging, molecular genomics, and population-level outcomes, converging evidence indicates that a subset of T1 lesions harbors aggressive, metastatically competent biology. Future management must therefore integrate biological risk, rather than depth alone, to refine staging, guide treatment selection, and identify patients who may benefit from systemic therapy.

## Data Availability

The data supporting the findings of this review are available within the article and its cited references.
